# Distinct downstream signaling and the roles of VEGF and PlGF in high glucose-mediated injuries of human retinal endothelial cells in culture

**DOI:** 10.1038/s41598-019-51603-0

**Published:** 2019-10-25

**Authors:** Wanzhen Jiao, Jia-Fu Ji, Wenwen Xu, Wenjuan Bu, Yuanjie Zheng, Aihua Ma, Bojun Zhao, Qingfeng Fan

**Affiliations:** 1 Department of Ophthalmology, Shandong Provincial Hospital Affiliated to Shandong University, Jinan, 250021 China; 2 Department of Anesthesiology, Affiliated Hospital of Shandong University of Traditional Chinese Medicine, Jinan, 250014 China; 3 Department of Ophthalmology, Jining First People’s Hospital, Jining, 272011 China; 4 School of Information Science and Engineering of Shandong Normal University, Jinan, 250014 China; 5 Institute of Biomedical Sciences of Shandong Normal University, Jinan, 250014 China; 6 Key Lab of Intelligent Computing & Information Security of Shandong Normal University, Jinan, 250014 China; 7 Shandong Provincial Key Laboratory for Distributed Computer Software Novel Technology, Jinan, 250014 China; 8 Department of Pediatrics, Shandong Provincial Hospital Affiliated to Shandong University, Jinan, 250021 China; 9 Renal-Electrolyte and Hypertension Division, Perelman School of Medicine of University of Pennsylvania, Philadelphia, PA 19104 USA

**Keywords:** Growth factor signalling, Molecular medicine

## Abstract

Vascular endothelial growth factor (VEGF) and placental growth factor (PlGF) plays a crucial role in breakdown of the blood-retinal barrier due to hyperpermeability in diabetic retinopathy (DR). However, the distinct signaling driven by VEGF and PlGF in the pathogenesis of DR remains unclear. In this study, we investigated VEGF- and PlGF- related signaling pathways and their roles in cultured human microvascular retinal endothelial cells (hRECs) under high glucose conditions (HG; 25 mM). Apoptotic cell death was evaluated, and FITC conjugated bovine serum albumin across monolayer hRECs served as an index of permeability. Western blots were used to assess the protein levels of VEGF and PlGF, as well as the phosphorylation of p38MAPK, STAT1 and Erk1/2. Knockdown of VEGF and PlGF was performed by using siRNA. Following HG treatment, increases of VEGF and PlGF as well as PKC activity were detected in hRECs. Increased phosphorylations of p38MAPK^Thr180/Thr182^, STAT1^Ser727^, and Erk1/2^Tyr202/Tyr185^ as well as VEGFR1^Tyr1213^ and VEGFR2^Tyr1175^ were also detected in HG-treated hRECs. Inhibition of PKC activity by Go 6976 prevented HG-induced increases of phosphor-Erk1/2 and nitric oxide synthase (NOS1) expressions as well as hyperpermeability, whereas inhibition of p38MAPK pathway by SB203580 selectively suppressed activation of STAT1 and decreased apoptotic cell death under HG conditions. Moreover, VEGF knockdown predominantly inhibited activation of VEGFR2, and phosphorylation of p38MAPK and STAT1, as well as apoptotic cell death in HG-treated hRECs. Nevertheless, PlGF knockdown mainly suppressed phosphorylation of VEGFR1, PKC, and Erk1/2, as well as NOS1 expressions and hyperpermeability. Taken together, we provide evidence demonstrating that HG-induced elevation of PlGF is responsible for hyperpermeability mainly through increasing activation of PKC-Erk1/2-NOS axis *via* VEGFR1, while HG-induced elevation of VEGF is associated with induction of apoptotic cell death mainly through increasing activation of p38MAPK/STAT1 signaling *via* VEGFR2.

## Introduction

Diabetic retinopathy (DR), one of the most prominent microvascular complications of diabetes mellitus, is the leading cause of new-onset blindness in the developed countries^1,2^. It was reported that across China, the prevalence of DR and sight-threatening DR was 27.9% and 12.6% in diabetes, respectively^3^. DR is classified as either non-proliferative DR, which is characterized by microaneurysms and intraretinal hemorrhage, or proliferative DR, which has been identified by vitreous hemorrhage and neovascularization of the eye fundus and iris^1^. Generally, it has been accepted that angiogenesis and inflammation crosstalk play an essential role in the pathogenesis of DR^4–6^. Diabetes and hyperglycemia have obvious effects on vascular endothelial cell permeability, adhesion and proliferation. Increased permeability results in vascular leakage and occlusions, as well as angiogenesis^4–6^.

Angiogenesis is a complex process that is mediated by various growth factors including vascular endothelial growth factor (VEGF) family^7^. VEGF family consists of seven secreted dimeric proteins, VEGF-A, B, C, D, E, F and the placental growth factor (PlGF). VEGF binds to at least two transmembrane tyrosine kinase receptors, named Flt1 (VEGF receptor-1 or VEGFR1) and Flk-1/KDR (VEGF receptor-2 or VEGFR2), on endothelial cells^8^. The levels of aqueous and vitreous VEGF-A were significantly elevated in patients with proliferative DR compared to the non-proliferative DR^9^. Unlike VEGF-A, VEGF-B possesses low angiogenic potential and does not induce vessel formation or sprouting. Signaling *via* VEGFR1 and neutropilin 1 (NRP-1), VEGF-B was considered as a potent survival factor of vascular cells which keeps the neo-vessels from apoptosis^10,11^. PlGF, a member belonging to the VEGF family, was originally isolated from the human placenta and directly signals through VEGFR1. It was reported that the levels of PlGF were elevated in the vitreous and aqueous humor of patients with DR^12,13^. A comparative research of vitreous PlGF levels in proliferative DR patients treated with or without anti-VEGF agent therapy revealed that PlGF levels were highly correlated with VEGF-A levels in active proliferative DR. This suggested that PlGF may also involve angiogenesis in the pathogenesis of DR possibly by amplifying the role of VEGF-A^14^. Some studies showed that stimulation of monocytes with PlGF or VEGF-A induced activation of several intracellular signaling molecules including phosphatidylinositol-3 kinase (PI3K), protein kinase B (Akt), extracellular signal-regulated kinase-1/2 (Erk1/2), and p38 mitogen-activated protein kinases (MAPK)^15–17^. Overproduction of nitric oxide synthase (NOS) induced by activated PKC is related to vasodilation and hyperpermeability^18^. STAT1 (signal transducer and activator of transcription 1) has been implicated as a mediator of a variety of biological responses such as apoptosis in response to stimulations of specific growth factors and cytokines^19^. However, the exact roles of VEGF and PlGF, and their distinct downstream signaling have not been understood completely in the pathogenesis of angiogenesis of DR.

The present study aims to investigate the distinct signaling pathways and their roles of VEGF and PlGF in high glucose (HG)-induced injuries of human microvascular retinal endothelial cells (hRECs). We demonstrated that in HG-treated hRECs, i) the abundances of both VEGF and PlGF were increased significantly, ii) VEGF-mediated activation of p38MAPK/STAT1 signaling *via* selectively binging to VEGFR2 mainly led to induction of apoptosis, and iii) PlGF-induced activation of PKC/Erk1/2/NOS1 pathway *via* selectively binding to VEGFR1 mainly resulted in hyperpermeability.

## Results

### Injuries of hREC in high glucose conditions

**Figure 1:**
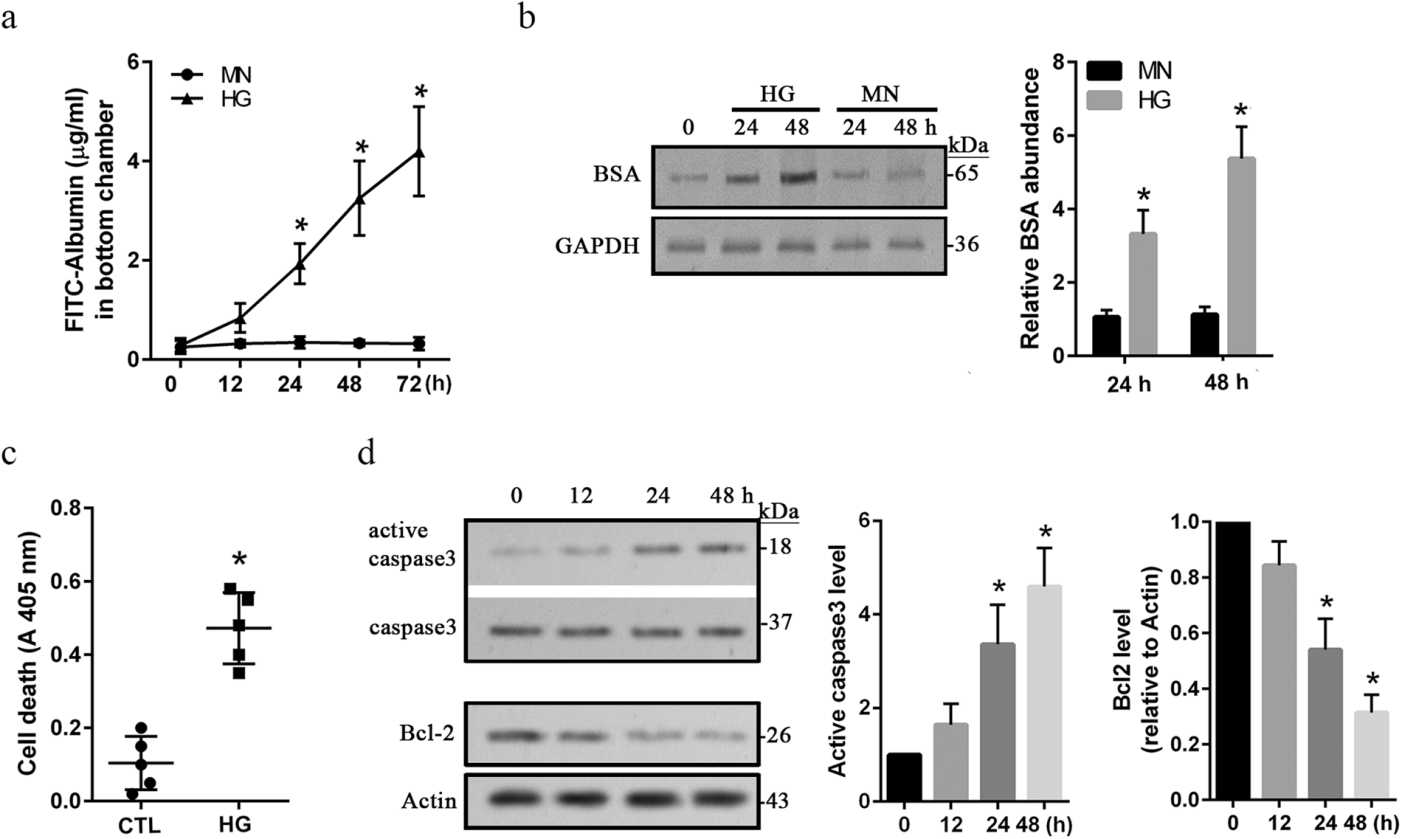
HG induces injuries in *in vitro* cultured hRECs. (**a**). hRECs were grown on Transwell filters. Cells were then treated with high glucose (HG, 25 mM) or mannitol (MN) as the osmotic control for different time periods. The permeability of monolayer cells was evaluated using FITC-conjugated albumin. *Data are presented mean ± SD*. *n* = *3*. *p < *0*.*05 vs 0 h*. (**b**). hRECs were grown in 6-well plate, and treated for 24 and 48 h with HG or MN, respectively. 10 μg/ml of bovine serum albumin (BSA) was then added and incubated for 30 min. After 3 washes, cells were lysed and total protein was extracted for immunoblotting with anti-BSA antibody. *Data are presented mean* ± *SD*. *n* = *3*. *p < *0*.*01 vs MN*. (**c**). hRECs were treated for 24 h with HG. Apoptotic cell death was determined by using the Cell Death Detection ELISA. *Data are presented mean ± SD*. *n = 5*. *: p < *0*.*05 vs control (CTL)*. (**d**). hRECs were treated with HG for the indicated time periods. Total cellular protein was extracted, and immunoblotting assay was performed for detection of activated caspase 3 and Bcl-2. MN-treated hRECs were used as the treatment control (Supplementary Fig. 3). *Data are presented mean* ± *SD*. *n* = *3*. *p < *0*.*01 vs 0 h*. *Full length blots of b and d are provided in* Supplementary Fig. 4.

A key manifestation of DR is macular edema which is mainly caused by increased microvascular permeability^20^. In this study, the permeability of monolayer hRECs growing on Transwell filters was evaluated by using FITC-conjugated bovine serum albumin (BSA). Compared to the mannitol (MN) osmotic control, HG caused a time-dependent increase of permeability, showing a significant (*p* < *0*.*05*) hyperpermeability at 24 h, persisting to 72 h (Fig. 1a). We also evaluated the uptake of BSA by using western blot assay. The amounts of BSA were increased significantly (*p* < *0*.*05*) at 24 and 48 h in the cellular lysates isolated from HG but not MN treated hRECs (Fig. 1b). These findings demonstrated that hyperpermeability of hRECs was induced under HG conditions. In addition, as reported previously^21^, increased apoptotic cell death was remarkably (*p* < *0*.*05*) detected at 48 h in HG treated cells in comparison with the controls (Fig. 1c). Moreover, increased active caspase3 level was detected significantly (*p* < *0*.*05*) at 24 and 48 h, whereas the anti-apoptotic protein Bcl-2 showed a significant (*p* < *0*.*05*) reduction in HG treated hRECs (Fig. 1d). Therefore, these findings suggest that injuries of hRECs under HG conditions present with increased apoptosis and hyperpermeability.

### Activation of the PKC-Erk1/2-NOS1 axis is related to hyperpermeability in HG-treated hRECs

**Figure 2:**
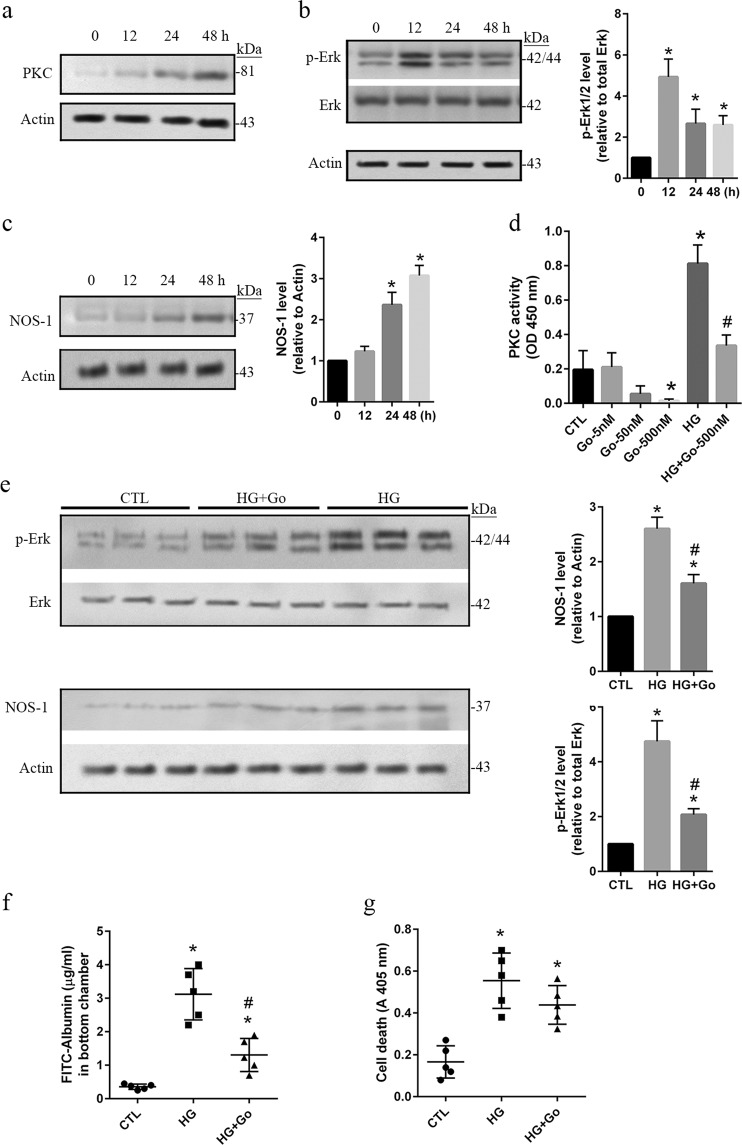
Activation of the PKC-Erk1/2-NOS1 axis is related to hyperpermeability in HG-treated hRECs. (**a**–**c**) hRECs were treated with high glucose (HG, 25 mM) as the indicated time periods. MN-treated hRECs were used as the treatment control (Supplementary Fig. 5a). Cells were then lysed and total protein was extracted for immunoblotting with anti-PKC (**a**), anti-phospho-Erk1/2^Tyr202/Tyr185^ (**b**) and anti-NOS1 (**c**) antibodies. *Data are presented mean* ± *SD*. *n* = *3*. *p < *0*.*05 vs 0 h*. (**d**). hRECs were cultured for 24 h under HG conditions in the presence or absence of PKC inhibitor Go 6976. Total cellular protein was extracted, and 50 μg was used for evaluation of PKC kinase activity. *Data are presented mean* ± *SD*. *n* = *3*. *p < *0*.*05 vs CTL;* ^*#*^*p* < *0*.*05 vs HG*. (**e**). hRECs were cultured for 24 h in HG with or without Go 6976 (500 nM). Cells were then lysed and total protein was extracted for immunoblotting with anti-phospho-Erk1/2^Tyr202/Tyr185^ and anti-NOS1 antibodies. The effects of Go 6976 on Erk activation were explored in hRECs under normoglycemia condition (Supplementary Fig. 5b). *Data are presented mean ± SD*. *n = 3*. *p < *0*.*05 vs CTL*. (**f**). Cells were grown on Transwell filters, and treated for 24 h in HG with or without Go 6976 (500 nM). The permeability of monolayer cells was evaluated using FITC conjugated albumin. (**g**). hRECs were cultured for 24 h in HG with or without Go 6976 (500 nM). Apoptotic cell death was determined using the Cell Death Detection ELISA. *f and g*. *Data are presented mean* ± *SD*. *n* = *5*. *p < *0*.*01 vs CTL;* ^*#*^*p* < *0*.*05 vs HG*. *Full length blots of a*, *b*, *c and e are provided in* Supplementary Fig. 6. *In b and e*, *stripped membranes from phospho-Erk were reblotted for total Erk*.

In HG-treated hRECs, the protein level of protein kinase C (PKC) was increased significantly (*p* < *0*.*05*) at 24 and 48 h (Fig. 2a). Following HG treatment, time-dependent increases of phospho-Erk1/2 at Tyr202/Tyr185 and NOS1 expression were detected (*p* < *0*.*05*), respectively (Fig. 2b,c). To investigate the role of PKC, Go 6976, a specific inhibitor of PKC activity was applied at 3 different concentrations. In normal cultured hRECs, the application of 500 nM of Go 6976 almost completely suppressed activation of PKC at 48 h. In this study, an increase of PKC activity was significantly (*p* < *0*.*05*) detected at 48 h in HG-treated hRECs, which was significantly (*p* < *0*.*05*) suppressed by Go 6976 at the concentration of 500 nM (Fig. 2d).

Interestingly, western blot assay revealed that HG-induced increases of phospho-Erk1/2^Tyr202/Tyr185^ and NOS1 expression at 48 h were significantly (*p* < *0*.*05*) inhibited by the application of 500 nM Go 6976. But, the phosphorylated Erk1/2 level and NOS1 expression were still higher (*p* < *0*.*05*) in HG-treated hRECs with Go 6976 than the controls (Fig. 2e). This suggests that HG mediated activation of Erk1/2-NOS1 signaling partially through upregulation of PKC activity. We also studied the effects of inhibition of PKC activity on permeability and apoptosis of hRECs under HG conditions. The presence of 500 nM Go 6976 significantly (*p* < *0*.*05*) decreased the permeability of FITC-albumin, but did not affect apoptotic level in hRECs treated with HG for 48 h (Fig. 2f,g). These findings demonstrate that overactivation of the PKC-Erk1/2-NOS1 signaling plays a critical role in regulating the permeability of hRECs under HG conditions.

### Activation of the p38MAPK/STAT1 pathway is related to apoptosis induction in HG-treated hRECs

**Figure 3:**
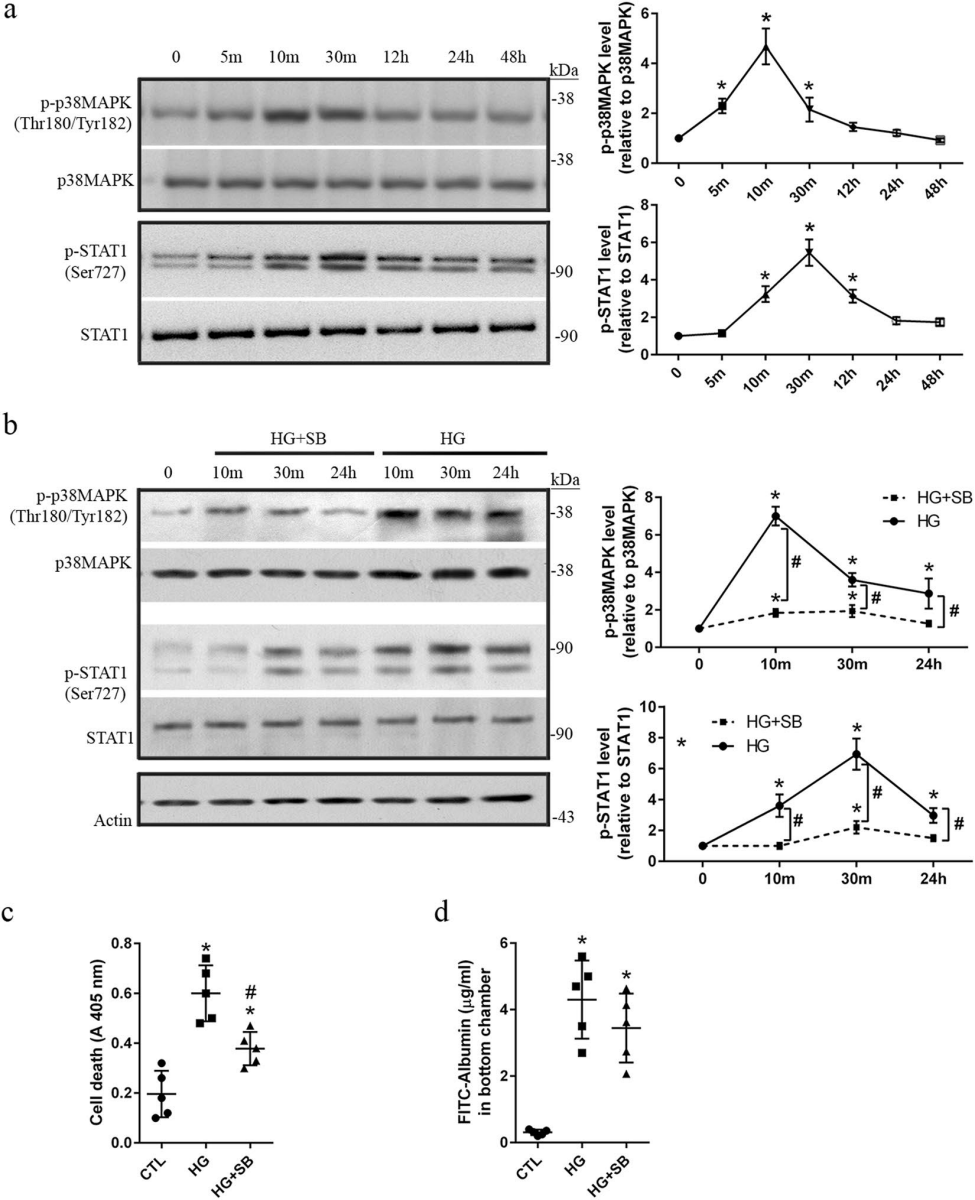
Activation of the p38MAPK/STAT1 signaling mediates apoptosis in HG-treated hRECs. (**a**). hRECs were treated with high glucose (HG, 25 mM) for the indicated time periods. Cells were lysed and total protein was extracted for immunoblotting with anti-phospho-p38 MAPK^Tyr180/Tyr182^ and anti-phospho-STAT1^Ser727^. MN-treated hRECs were used as the treatment control (Supplementary Fig. 7a). *Data are presented mean* ± *SD*. *n* = *3*. *p < *0*.*05 vs 0 h*. (**b**). hRECs were treated for the indicated time periods with HG in the presence or absence of p38 MAPK inhibitor SB203580 (10 μM). Cells were lysed and total protein was extracted for immunoblotting with anti-phospho-p38MAPK^Tyr180/Tyr182^ and anti-phospho-STAT1^Ser727^. The effects of SB203580 on STAT1 activation were explored in hRECs under normoglycemia condition (Supplementary Fig. 7b). *Data are presented mean ± SD*. *n = 3*. *p < *0*.*05 vs 0 h;* ^*#*^*p* < *0*.*05*, *HG vs HG + SB*^.^ (**c**). hRECs were treated for 24 h with HG in the presence or absence of SB203580 (10 μM). Apoptotic cell death was determined using the Cell Death Detection ELISA. (**d**). Cells were grown on Transwell filters and treated for 24 h with HG in the presence or absence of SB203580 (10 μM). The permeability of monolayer cells was evaluated using FITC conjugated albumin. *c and d: Data are presented mean ± SD*. *n = 5*. *p < *0*.*05 vs CTL;* ^*#*^*p* < *0*.*05 vs HG*. *Full length blots of a and b are provided in* Supplementary Fig. 8. *In a and b*, *stripped membranes from phospho-p38MAPK and phosphor-STAT1 were reblotted for total p38MAPK and STAT1*, *respectively*.

It has been well known that activation of the p38MAPK signaling plays an important role in inducing apoptosis in various cell types^22^. In this study, rapid increase of phospho-p38MAPK at Thr180/Tyr182 was significantly (*p* < *0*.*05*) detected at 5, 10, and 30 min following HG treatment. In comparison with the dynamics of p38MAPK activation, a delay increase of phospho-STAT1 at Ser727 was significantly (*p* < *0*.*05*) revealed at 10 and 30 min in HG-treated hRECs (Fig. 3a). SB203580, a specific p38MAPK inhibitor, was applied to inhibit activation of p38MAPK signaling. HG-induced increase of phosphorylated p38MAPK was significantly (*p* < *0*.*05*) prevented by the application of 10 µM SB203580 **(**Fig. 3b**)**. It was reported that p38MAPK is able to phosphorylate STAT1 at Ser727 *in vitro*^23^. As expected, upregulation of phosphorylated STAT1 was significantly (*p* < *0*.*05*) inhibited by 10 µM SB203580 in HG-treated hRECs (Fig. 3b). We also explored the effects of p38MPAK inhibition on permeability and apoptosis in HG-treated hRECs. Interestingly, SB203580 remarkably (*p* < *0*.*05*) decreased HG-induced apoptotic cell death, but did not show effect on hyperpermeability of FITC-albumin (Fig. 3c,d). These findings show that overactivation of the p38MAPK/STAT1 pathway is required at least in part for induction of apoptotic cell death of hRECs under HG conditions.

### Abundances of VEGF and PlGF are increased in HG-treated hRECs

**Figure 4:**
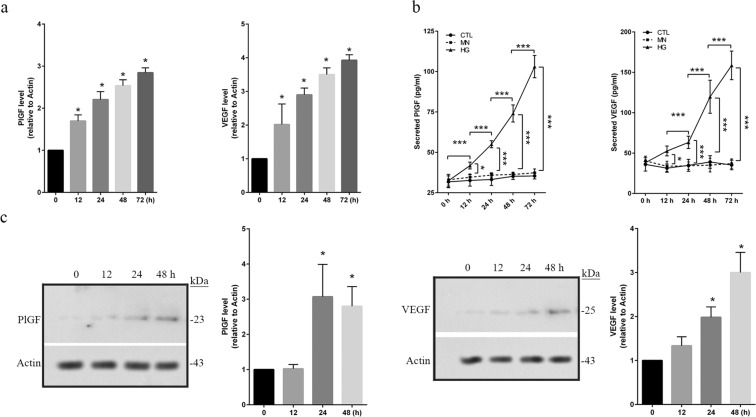
The abundances of PlGF and VEGF are increased in HG-treated hRECs. hRECs were treated with high glucose (HG, 25 mM) for the indicated time periods. Non-treated cell (CTL) or mannitol (MN) treated cell was used as the controls. (**a**). Quantitative real PCR was performed to assess the mRNA levels of VEGF and PlGF. (**b**). Culture medium was collected and ELISA was performed to quantitate the amount of secreted PlGf and VEGF, respectively. (**c**). Cells were lysed and total protein was extracted for immunoblotting with anti-PlGF and anti-VEGF antibodies. Full length blots are shown for PlGF and VEGF. MN-treated hRECs were used as the treatment control (Supplementary Fig. 9). *Data are presented mean ± SD*. *n = 3*. b: *p < *0*.*05*, **p < *0*.*01*, ***p < *0*.*001*. *n*.*s*. *non-significance*. a and c: *p < *0*.*01 vs 0 h*.

Here, the abundances of VEGF and PlGF were measured at different time points in HG-treated hRECs. The mRNA levels of VEGF and PlGF were quantitatively assessed by using real time RT-PCR, showing a time-dependent increase (*p* < *0*.*05*) starting at 12 h in HG-treated hRECs (Fig. 4a). We also measured the amounts of secreted VEGF and PlGF by using ELISA assay in the cultured media. Significant increase was detected (*p* < *0*.*05*) since 12 h following HG treatment (Fig. 4b). Moreover, the abundances of VEGF and PlGF proteins were significantly (*p* < *0*.*05*) upregulated at 24 and 48 h following HG treatment (Fig. 4c). These data suggest that both expressions and secretions of VEGF and PlGF were remarkably increased under HG conditions.

### VEGF predominantly activates the p38MAPK/STAT1 signaling *via* VEGFR2 and thus induces apoptotic cell death in HG-treated hRECs

**Figure 5:**
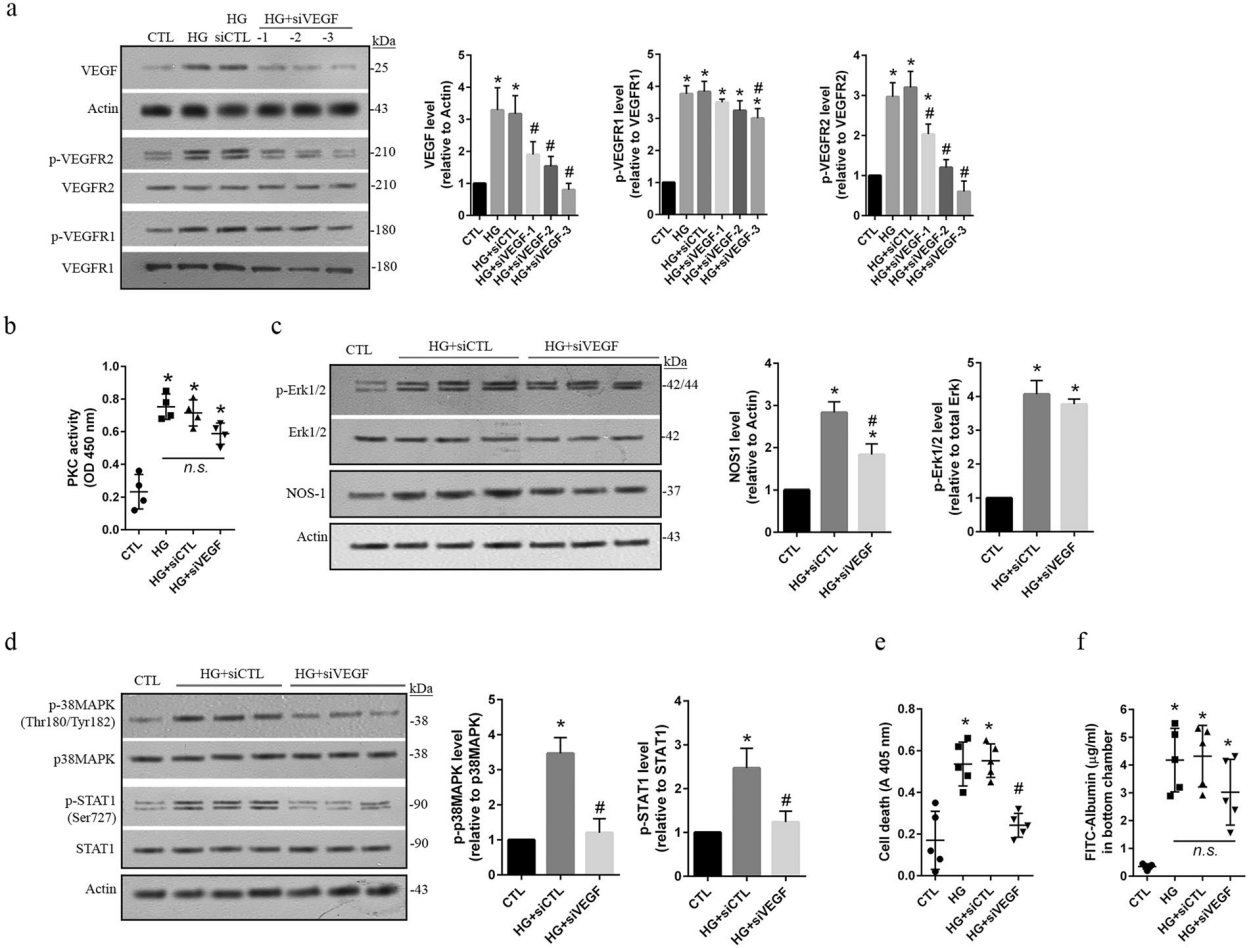
VEGF-mediated activation of the p38MAPK/STAT1 signaling via VEGFR2 involves increase of apoptosis in HG-treated hRECs. (**a**). In cultured hRECs, the siRNA that targets to human *VEGFA* gene was introduced at 3 different concentrations (siVEGF-1: 5 nM, siVEGF-2: 10 nM, siVEGF-3: 20 nM). Control siRNA (siCTL, 20 nM) that does not target any human gene was used as the transfection control. 24 h after transfection, cells were exposed to high glucose (HG, 25 uM) for 24 h. Cells were then lysed and total cellular protein was extracted for immunoblotting with anti-VEGF, anti-phospho-VEGFR1^Tyr1213^, and anti-phospho-VEGFR2Tyr1175 antibodies. (**b**). In VEGF knockdown cell (siVEGF, 20 nM), effect of HG on PKC activity was evaluated using PKC Kinase Activity Assay. (**c**). In VEGF knockdown cell (siVEGF, 20 nM), immunoblot assay was performed to investigate the effects of HG on the level of phospho-Erk1/2^Tyr202/Tyr185^ and NOS1 expression. (**d**). In VEGF knockdown cell (siVEGF, 20 nM), immunoblot assay was performed to show the effects of HG on the level of phospho-p38MAPK^Tyr180/Tyr182^ and phospho-STAT1^Ser727^. (**e**). In VEGF knockdown cell (siVEGF, 20 nM), apoptotic cell death was determined following HG treatment. (**f**). Cells were grown on Transwell filters, and VEGF was knocked down using siVEGF (20 nM). Following HG treatment, the permeability of monolayer cells was measured. *Data are presented mean ± SD*. *a*, *c and d: n = 3; B: n = 4; e and f: n = 5*. *p < *0*.*05 vs CTL*, ^*#*^*p* < *0*.*05 vs HG or HG + siCTL; n*.*s*. *non-significance*. *In c and d*, *stripped membranes from phospho-Erk*, *phospho-p38MAPK*, *and phospho-STAT1 were reblotted for total Erk*, *p38MAPK*, *and STAT1*, *respectively*.

VEGF family and its receptors are vitally involved in the process of angiogenesis^8^. To specify the downstream signaling of VEGF, we deleted VEGF expressions by using 3 different concentrations of siRNA that is specifically targeted to human VEGF-A. Western blot assay shows a significant (*p* < *0*.*05*) dose-dependent reduction of VEGF in HG-treated hRECs expressing siVEGF-A (Fig. 5a). Interestingly, the levels of both phospho-VEGFR1 at Tyr1213 and phospho-VEGFR2 at Tyr1175 were significantly (*p* < *0*.*05*) increased following HG-treatment. However, increased phospho-VEGFR2^Tyr1175^, but not phospho-VEGFR1^Tyr1213^, was significantly (*p* < *0*.*05*) inhibited by all 3 concentrations of siVEGF-A (Fig. 5a). Increase of the phosphorylated VEGFR1^Tyr1213^ level was slightly inhibited in HG-treated hRECs that only expressed high concentrations of siVEGF-A. These data suggest that in cultured hRECs exposed to HG conditions, increased VEGF mainly activated VEGFR2. In addition, knockdown of VEGF did not have significant (*p > 0*.*05*) influence on PKC activity in HG-treated hRECs (Fig. 5b). Moreover, HG-induced increases of phospho-Erk1/2^Tyr202/Tyr185^ were not affected by VEGF knockdown (Fig. 5c). The level of NOS1 was significantly (*p* < *0*.*05*) decreased in HG-treated hRECs expressing siVEGF-A compared to HG-treated cells expressing siCTL, although it was still higher than nontreated cells (Fig. 5c). However, HG-increased phospho-p38MAPK^Tyr180/Tyr182^ and phospho-STAT1^Ser727^ was significantly (*p* < *0*.*05*) suppressed by VEGF knockdown (Fig. 5d). In consistent, VEGF knockdown significantly (*p* < *0*.*05*) decreased apoptotic cell death (Fig. 5e), but did not show obvious effects on permeability in HG-treated hRECs (Fig. 5f). These findings demonstrate that VEGF selectively activated VEGFR2 by enhancing its phosphorylation at Tyr1175, thus inducing apoptotic cell death through p38MAPK/STAT1 signaling pathway.

### PlGF mainly activates the PKC-Erk1/2-NOS1 axis *via* VEGFR1 responsible for hyperpermeability in HG-treated hRECs

**Figure 6:**
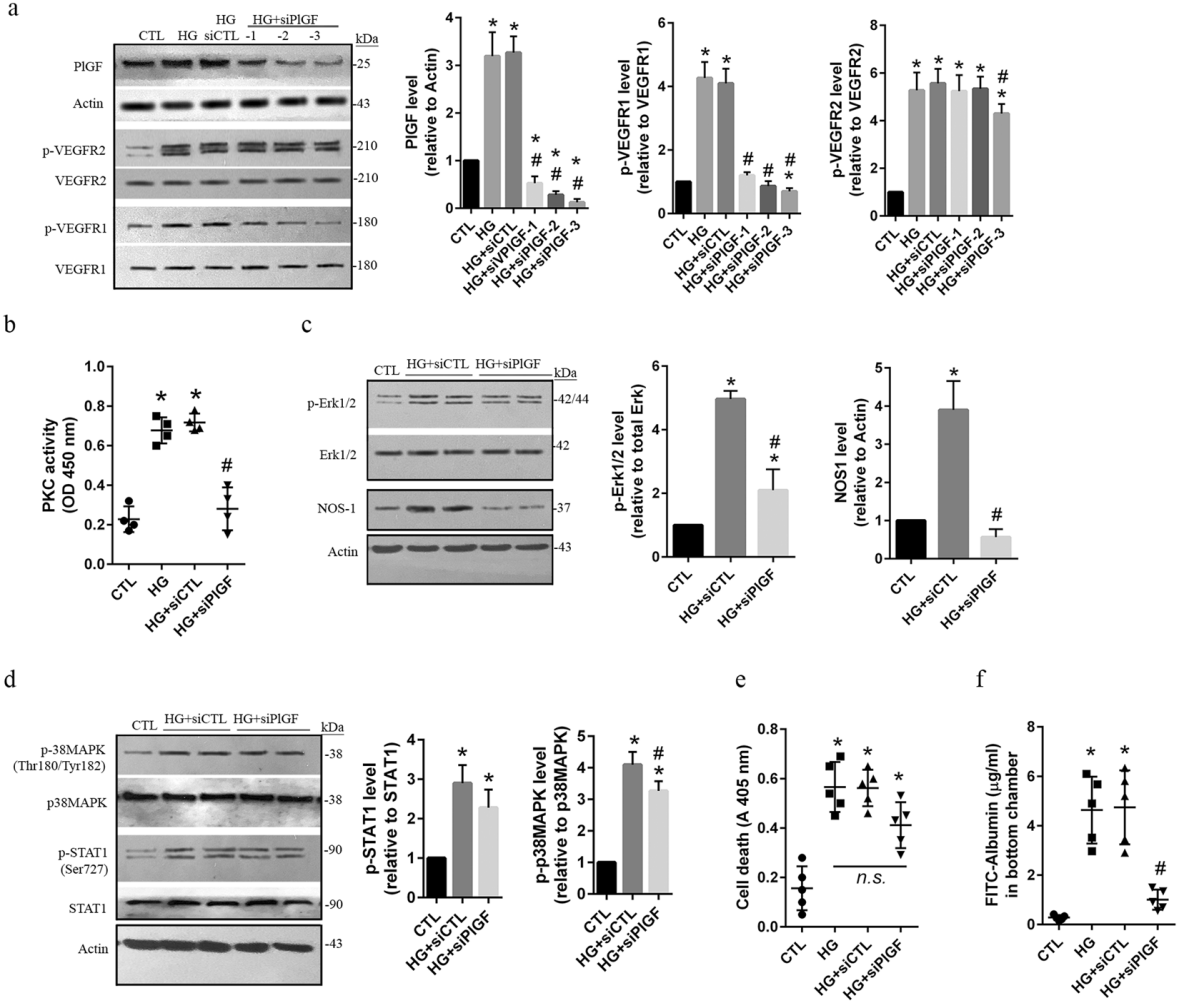
PlGF-mediated activation of the PKC-Erk1/2-NOS axis via VEGFR1 is mainly related to increase of permeability in HG-treated hRECs. (**a**). In cultured hRECs, the siRNA that targets to human *PlGF* gene was introduced at different concentration (siPlGF-1: 5 nM, siPlGF-2: 10 nM, siPlGF-3: 20 nM). Control siRNA (siCTL, 20 nM) that does not target any human gene was used as the transfection control. 24 h after transfection, cells were exposed to high glucose (HG, 25 uM) for 24 h. Cells were then lysed and total cellular protein was extracted for immunoblotting with anti-PlGF, anti-phospho-VEGFR1^Tyr1213^, and anti-phospho-VEGFR2^Tyr1175^ antibodies. (**b**). In PlGF knockdown cell (siPlGF, 20 nM), effect of HG on PKC activity was evaluated using PKC Kinase Activity Assay. (**c**). In PlGF knockdown cell (siPlGF, 20 nM), immunoblot assay was performed to investigate the effects of HG on the level of phospho-Erk1/2^Tyr202/Tyr185^ and NOS1 expression. (**d**). In PlGF knockdown cell (siPlGF, 20 nM), immunoblot assay was performed to show the effects of HG on the level of phospho-p38 MAPK^Tyr180/Tyr182^ and phospho-STAT1^Ser727^. (**e**). In PlGF knockdown cell (siPlGF, 20 nM), apoptotic cell death was determined following HG treatment. (**f**). Cells were grown on Transwell filters, and PlGF was knocked down using siPlGF (20 nM). Following HG treatment, the permeability of monolayer cells was measured. *Data are presented mean ± SD*. *a*, *c and d: n = 3; b: n = 4; e and f: n = 5*. *p < *0*.*05 vs CTL*, ^*#*^*p* < *0*.*05 vs HG or HG + siCTL; n*.*s*. *non-significance*. *In c and d*, *stripped membranes from phospho-Erk*, *phospho-p38MAPK*, *and phospho-STAT1 were reblotted for total Erk*, *p38MAPK*, *and STAT1*, *respectively*.

PlGF belongs to the VEGF family, and its level was elevated in the vitreous and aqueous humor of patients with DR^12–14^. Here, knockdown of PlGF was obtained by introducing 3 different concentrations of siPlGF specifically targeted to human PlGF in HG-treated hRECs. The results showed that HG-induced increase of PlGF was dramatically (*p* < *0*.*05*) suppressed by siPlGF (Fig. 6a). We then investigated the effects of PlGF knockdown on phosphorylated VEGFR1 and phosphorylated VEGFR2 level. Our data show that increased phospho-VEGFR1^Tyr1213^, but not phospho-VEGFR2^Tyr1175^, was remarkably (*p* < *0*.*05*) inhibited by knockdown of PlGF in HG-treated hRECs compared to HG alone and HG-treated cells with siCTL (Fig. 6a). This suggests that PlGF may predominantly activate VEGFR1 following HG stimulations in hRECs. Knockdown of PlGF showed significant (*p* < *0*.*05*) inhibition on PKC activity in HG-treated hRECs (Fig. 6b). Moreover, HG-induced increases of phospho-Erk1/2^Tyr202/Tyr185^ and NOS1 expressions were significantly (*p < 0*.*05*) suppressed by PlGF knockdown (Fig. 6c). Compared to nontreated cells, the level of phosphorylated Erk1/2 was still higher (*p* < *0*.*05*) in HG-treated hRECs with PlGF knockdown (Fig. 6c), suggesting that other signal pathway may involve Erk1/2 activation in hRECs under HG conditions. However, increased phospho-p38MAPK^Tyr180/Tyr182^ and phospho-STAT1^Ser727^ was not affected by PlGF knockdown in HG-treated hRECs (Fig. 6d). In consistent, PlGF knockdown did not show obvious effects on apoptosis (Fig. 6e); but, significantly (*p* < *0*.*05*) decreased permeability in HG-treated hRECs (Fig. 6f).

## Discussion

Proliferative DR is the advanced stage of DR, characterized by retinal neovascularization and hyperpermeability, hemorrhaging pre-retinal neovessels and potential vitreal contraction, and retinal detachment^24,25^. Deterioration of the blood-retinal barrier results in extravasation of fluids from permeable capillaries, leading to diabetic macular edema^10^. It has been reported that several factors including elevated levels of VEGF and PlGF involve breakdown of the blood-retinal barrier in DR^10^. In the present study, we demonstrated that in cultured hRECs under HG conditions, elevated VEGF and PlGF significantly increased endothelial cell permeability and apoptotic cell death by distinct downstream signaling PKC/Erk/NOS and p38MAPK/STAT1 pathways, respectively. A potential concern is whether PlGF-induced hyperpermeability results from a loss of cells under HG condition. Actually, we did not observe significant detachment of hRECs following HG treatment. Moreover, confluent monolayer cells were used for Transwell assay, and the western blot assay also detected increased BSA uptake in HG-treated hRECs. Therefore, PlGF-induced hyperpermeability may not result from a loss of cells in HG-treated hRECs. However, it should be noted here that the FITC-BSA and Transwell assays may not be sufficient to assess the endothelial permeability. More accurate methods such as trans-endothelial electrical resistance (TEER) and electrical cell-substrate impedance sensing (ECIS) should be used in the future for permeability analysis.

As described previously^26^, we detected increases of the mRNA and protein levels of VEGF and PlGF as well as the concentrations of secreted VEGF and PlGF in HG-treated hRECs. Binding of VEGF to VEGFR2 led to activation of PI3K and phospholipase C-γ, which then activated PKC and the release of free calcium from internal stores^27^. In this study, increased activation of PKC was detected in cultured hRECs under HG conditions. Inhibition of PKC activation predominantly decreased permeability; but, did not have remarkable influences on apoptotic cell death. Studies with specific inhibitors revealed a role for the ERK1/2 in VEGF-induced hyperpermeability^28^. Growth factors are thought to activate ERK1/2 through the Ras-Raf-MEK pathway^29^. However, it was found that VEGF can induce Ras-independent ERK1/2 activation in which PKC involved^30^. Here, increased phosphorylation of Erk1/2 and NOS1 expressions were detected in HG-treated hRECs, which was prevented by the inhibition of PKC. Increased activity of PKC can result in activation of endothelial NOS, leading to NO release^31^. It has been reported that increased NO is related to vasodilation and hyperpermeability^27^. In cultured human umbilical vein endothelial cell, ERK1/2 and NOS are elements of different signaling pathways that are associated with VEGF-induced hyperpermeability^32^. Therefore, our findings suggest that HG-induced hyperpermeability may be mainly mediated by the activation of PKC-Erk1/2-NOS axis in cultured hRECs.

Under HG conditions, it has been reported that p38MAPK and Erk1/2 is activated in hRECs^26,33,34^. Increased serine phosphorylation of transcription factor STAT1 followed by activation of p38MAPK plays an important role in apoptosis induction in HeLa contaminant WISH cell^35^. Here, increased phosphorylation of p38MAPK at Tyr180/Tyr182 and STAT1 at Ser127 were detected in HG-treated hRECs. Inhibition of p38MAPK significantly suppressed phospho-STAT1 level and apoptotic cell death; but did not show remarkable influences on permeability in HG-treated hRECs. These findings suggest that overactivation of p38MAPK/STAT1 signaling may preferably induce apoptotic cell death in HG-treated hRECs. In addition, as previously reported^26,33,34^, increased activation of Akt was also detected in our HG-treated hRECs (data not shown). However, inhibition of PKC and p38MAPK did not alter phospho-Akt level in HG-treated hRECs (data not shown), suggesting that Akt activation may be independent on PKC and p38MAPK signaling pathway.

**Figure 7:**
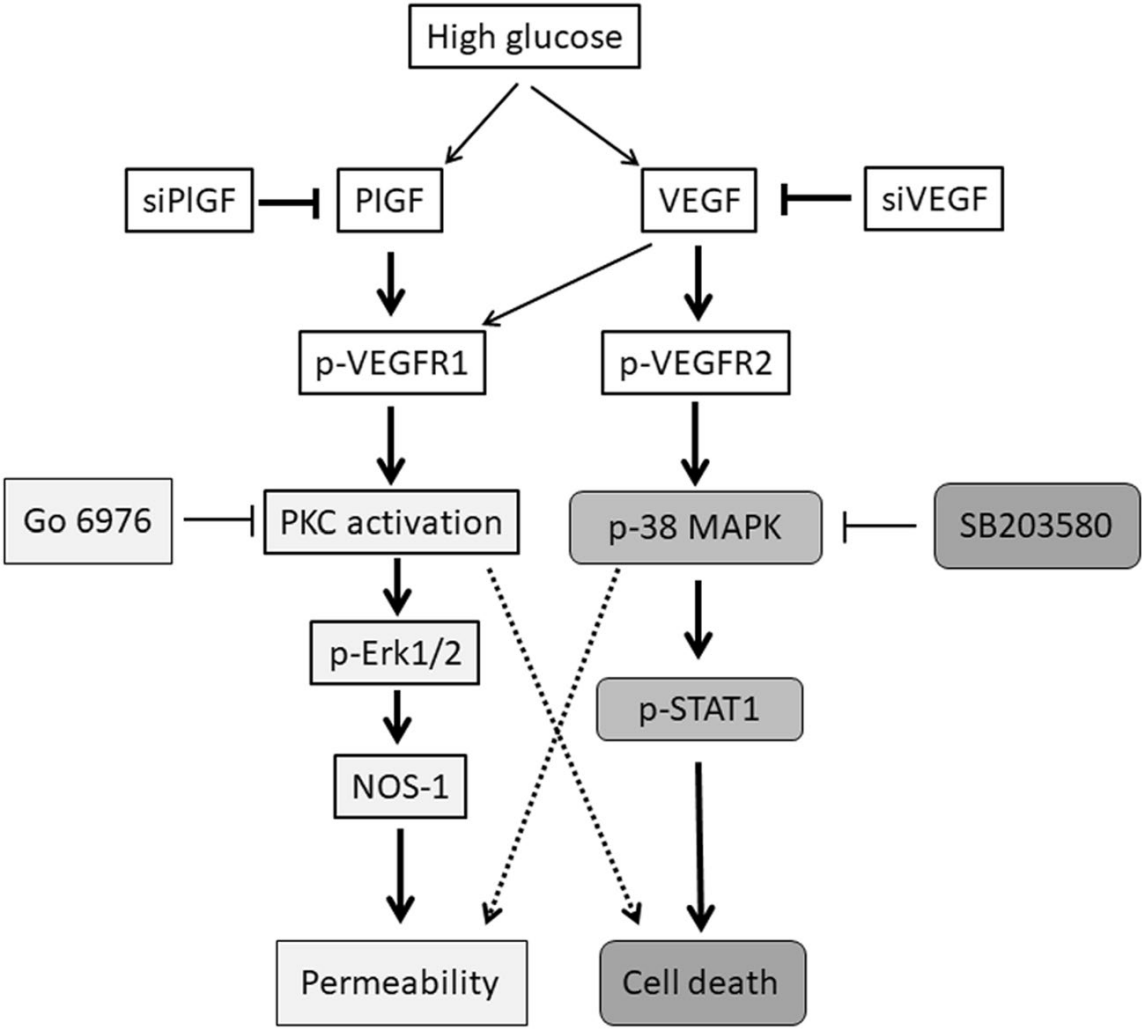
The schematic of the proposed VEGF and PlGF signaling in HG-induced hRECs injuries. Hyperpermeability and increased cell death was detected in HG-treated hRECs. Following HG treatment, expression of VEGF and PlGF was upregulated, phosphorylation of VEGFR1 and VEGFR2 as well as PKC activity and NOS1 abundance were increased, and activation of Erk1/2, p38MAPK and STAT1 were increased. Inhibition of PKC activity predominantly prevented activation of Erk1/2 and decreased NOS1 level and permeability, while did not affect apoptotic cell death. Inhibition of p38MAPK dramatically inhibited activation of STAT1 and decreased cell death, while did not show significant effect on permeability. Knockdown of VEGF mainly decreased phosphorylation of VEGFR2 and p38MAPK as well as cell death; knockdown of PlGF mainly decreased phosphorylation of VEGFR1 as well as PKC activity and permeability. *Go 6976: PKC inhibitor*, *SB203580: p38MAPK inhibitor*.

In hRECs exposed to VEGF and HG, VEGF upregulated PlGF expression *via* the MAPK signaling pathway and partially through PKC^36^. It was found that fibroblast growth factor (FGF2)-mediated enhancement of PlGF expression was dependent on VEGF function^37^. Therefore, a potential crosstalk is present between VEGF and PlGF signaling. To separate the distinct role of VEGF and PlGF in HG-induced injuries of hRECs, we firstly assessed the phosphorylation levels of VEGFR1 and VEGFR2, and then performed knockdown assay by using the siRNA that specifically targeted against VEGF-A and PlGF, respectively. Here, we detected significant upregulation of the phosphorylated VEGFR1 at Tyr1213 and VEGFR2 at Tyr1175 following HG treatment. Some studies reported that the expression of VEGFR1 and VEGFR2 is upregulated in the retinal vessels of diabetic rats^38^. However, our results show that knockdown of PlGF significantly decreased the tyrosine phosphorylation level of VEGFR1 but not VEGFR2 in HG-treated hRECs, suggesting that PlGF may mainly transduce downstream signaling *via* activation of VEGFR1 in cultured hRECs under HG conditions. The strong correlation of PlGF levels with proliferative DR disease status and expression of VEGFR1 was reported in human proliferative DR^14^. Interestingly, knockdown of PlGF dramatically prevented HG-induced increase of PKC activity. In placenta cell lines exposed to HG, the expressions of PlGF and VEGF as well as PKCβ activity were also increased significantly^39^. Here, we found that increased phospho-Erk1/2 and NOS1 expressions were significantly inhibited by knockdown of PlGF in HG-treated hRECs. Consistently, knockdown of PlGF decreased permeability; but, did not affect the level of apoptotic cell death under HG conditions. Our findings suggest that PlGF mainly increased the activation of PKC-Erk1/2-NOS axis *via* VEGFR1, thus leading to hyperpermeability in HG-treated hRECs **(**Fig. 7**)**. However, stimulations of immortalized bovine RECs with various growth factors showed that VEGF-A, but not PlGF, impairs the barrier function of bovine RECs, and that activation of VEGFR2, probably in concert with neuropilin-1, seems to be sufficient to induce barrier dysfunction^40^. Therefore, the addition of PlGF or VEGF to hRECs treated with or without HG may provide more important information. This could help us further understand the role and downstream signaling of PlGF and VEGF in retinal endothelial cell injuries.

Notably, only *in vitro* experiments were performed in this study since the current condition does not allow to do *in vivo* explorations. The data from human endothelial cultures are unlikely to address a complex *in vivo* issue that involves multiple cell-types. Therefore, it is well worth of investigating and validating the present findings in an *in vivo* DR model. Recently, increased PlGF and VEGF was detected in a new diabetic mouse strain, Akita, and PlGF deletion in these mice prevented diabetic-induced retinal cell death, capillary degeneration, pericyte loss, and blood-retinal barrier breakdown^41^. This suggests that PlGF is critical for the development of DR, and its genetic deletion protects the retina from diabetic damage. The conclusion is in consistent with our findings that HG increased both PlGF and VEGF expressions, and PlGF knockdown mainly prevented HG-induced hyperpermeability in hRECs. Nevertheless, we did not find that PlGF knockdown had significant effect on HG-induced cell death. Additionally, PlGF knockout inhibited the HIF1α-VEGF signaling pathway in the Akita mice retinas, presenting with reduction of phospho-VEGFR1, phospho-VEGFR2 and phospho-eNOS compared to wild type (WT) Akita mice^41^. In our *in vitro* model, PlGF knockdown predominantly decreased the levels of phospho-VEGFR1 and total NOS1, but not phospho-VEGFR2. The Akt pathway is a signal transduction pathway that promotes survival and growth in response to extracellular signals. Akt activity was reduced in the WT Akita mice, while increased dramatically in PlGF knockout Akita mice^41^. However, our and other studies^26,33,34^ showed a significant activation of Akt in HG-treated hRECs (data not shown). These discrepancies may be resulted from the differences between the *in vivo* and the *in vitro* experiments. The other thing is that global knockout of PlGF was obtained in the Akita mice, which may be the other potential reason for the discrepancies. Therefore, future work is needed to perform specific knockout of PlGF in the retinal endothelial cells in the Akita mice in order to determine the distinct role and downstream signaling of PlGF. Moreover, interaction of pericytes with endothelial cells is important for endothelial cell viability. The role and proximal signaling of VEGF and PlGF need be further investigated in HG-induced hRECs injuries in the presence of pericytes.In this study, activation levels of p38MAPK/STAT1 were significantly suppressed by knockdown of VEGF but not PlGF in HG-treated hRECs. Our data show that VEGF knockdown selectively inhibited activation of VEGFR2 in cultured hRECs under HG condition. HG-induced overactivation of p38MAPK/STAT1 signaling was significantly prohibited in VEGF knockdown cell. Nevertheless, VEGF knockdown showed no remarkable influences on PKC activity as well as phosphorylated Erk1/2 and NOS1 expressions in HG treated hRECs. In consistent, apoptotic cell death, but not hyperpermeability, was significantly decreased by VEGF knockdown in HG-treated hRECs. It was reported that increased VEGF and p38MAPK activation were also related to induction of apoptosis in hRECs under HG conditions^33^. Therefore, our findings suggest that VEGF predominantly increased the activation of p38MAPK/STAT1 *via* VEGFR2, thus leading to induction of apoptotic cell death in HG-treated hRECs **(**Fig. 7**)**.

Taken together, we separated the downstream signaling of VEGF and PlGF in HG-induced injuries of hRECs. In HG-treated hRECs, VEGF-mediated activation of p38MAPK/STAT1 signaling *via* binging to VEGFR2 mainly led to apoptosis, while PlGF-mediated activation of PKC-Erk1/2-NOS1 signaling *via* binding to VEGFR1 mainly resulted in hyperpermeability.

## Materials and Methods

### Primary antibodies

The following primary antibodies were used in this study: mouse anti-bovine serum albumin (B2901, 1: 1,000), rabbit anti-NOS1 (SAB4502010, 1: 500), and mouse anti-β-actin (A2228, 1: 10,000) antibodies from Sigma-Aldrich (St. Louis, MO, USA); rabbit anti-caspase3 (ab32351, 1: 1,000), rabbit anti-active caspase 3 (ab2302, 1: 500), rabbit anti-Bcl2 (ab59348, 1: 1,000), rabbit anti-PKC (ab19031, 1: 500), mouse anti-Erk1/2 (ab54230, 1: 1,000), rabbit anti-phospho-Erk1^Tyr202^ and Erk2^Tyr185^ (ab201015, 1: 1,000), rabbit-anti-Flit1/VEGFR1 (ab2350, 1: 500), rabbit anti-KDR/VEGFR2 (ab39256, 1: 600), rabbit anti-human CD31 (ab28364, 1: 250), rabbit anti-Von Willebrand Factor antibody (ab6994, 1: 250), and rabbit anti-PlGF (ab9542, 1: 500) antibodies from Abcam (Cambridge, MA, USA); mouse anti-VEGF (cat. no. 05–1117, 1: 1,000) and rabbit anti-phospho-VEGFR1^Tyr1213^ (cat. no. 07–758, 1: 500) antibodies from EMD Millipore (Burlington, MA, USA); rabbit anti-phospho-VEGFR2^Tyr1175^ (#2478, 1: 800), rabbit anti-phospho-p38MAPK^Thr180/Tyr182^ (#4631 S, 1: 1,000), rabbit anti-p38MAPK (#9212, 1: 1,000), rabbit anti-STAT1 (#9172, 1: 500), and rabbit anti-phospho-STAT1^Ser727^ (#8826, 1: 500) antibodies from Cell Signaling Technology (Danvers, MA, USA). Alexa Fluo 594 goat anti-rabbit IgG antibody (Cat. no. A-11037, 1: 2,000, Invitrogen; Thermo Fisher Scientific, Inc., Waltham, MA USA) was used for indirect immunofluorescence staining assay.

### Cell culture and treatment

Human microvascular retinal endothelial cell line (ACBRI 181, hRECs) was obtained from Cell Systems (Kirkland, WA, USA). As previously described^33^, hRECs were grown in fibronectin-coated plate in Dulbecco’s Modified Eagle’s Medium (cat. no. 11885–084) supplemented with 10% fetal calf serum (cat. no. 16000-044) and 100 U/ml of penicillin/streptomycin (cat. no. 15140-122; Gibco; Thermo Fisher Scientific, Inc.). To maintain uniform conditions, all experiments were performed by using passage 4–6 hRECs in this study. Cultured hRECs were also identified by stain of CD13 and von Willebrand Factor (vWF)^34^; the positive staining of CD13 and vWF has been provided **(**Supplemental Fig. 1).

hRECs were cultured in the presence of 25 mM of glucose (cat. no. G8270, Sigma-Aldrich) or mannitol (cat. no. PHR1007, Sigma-Aldrich) for different time periods. In some cases, 10 µg/ml bovine serum albumin (BSA; B8667, Sigma-Aldrich), 500 nM PKC inhibitor Go 6976 (cat. no. 2253/1, R&D Systems; Minneapolis, MN), and 10 µM p38 MAPK inhibitor SB203580 (#5633, Cell Signaling Technology) was added to the medium alone or in appropriate combination as indicated.

### Cell death detection

Apoptotic cell death was determined using the Cell Death Detection ELISA kit (cat. no. 11 774 425 001; Roche; Mannheim, Germany) as described previously^21^. Triplicate was used in each condition. The absorbance at 405 nm and 490 nm (reference wavelength) was determined with a microplate reader (Bio-Tec Instruments, Winooski, VT, USA). Signals in the wells containing the substrate only were used as the background and subtracted.

### ELISA assay

The amounts of VEGF and PlGF in cell culture supernatants were measured using Human VEGF ELISA Kit (ab100662, Abcam) and Human PlGF Quantikine ELISA Kit (cat. no. DPG00, R&D Systems) according to the manufacturer’s procedure, respectively.

### Permeability detection assay

hRECs were seeded on polyethylene membrane insert (a pore size of 1.0 μm) in the Transwell chamber and treated with HG or mannitol for the indicated time periods. FITC-conjugated bovine serum albumin (BSA; cat. no. A9771, Sigma-Aldrich) was added at the final concentration of 10 μg/ml in each upper chamber, whereas the lower chamber was filled with 500 μl medium alone. Transwell plates were incubated for 30 min at 37 °C. The solution in lower chamber containing permeabilized FITC-BSA was collected to measure the fluorescence intensity using microplate reader (Bio-Tec Instruments) with the excitation and emission wavelengths of 490 and 525 nm, respectively. The amount of permeabilized FITC-BSA (μg/ml) was then calculated according to the standard concentration curve.

### RT-PCR

Quantitative real time PCR was performed as described previously^21^. Briefly, total RNA was extracted and quantitated. Totally, 2 μg of RNA was transcribed to the cDNA with SuperScript™ III First-Strand Synthesis Kit (cat. no. 11752-050; Thermo Fisher Scientific, Inc.). Quantitative PCR was then performed using a 7500 fast Real-Time PCR system (Applied Biosystems; Thermo Fisher Scientific, Inc.). The SYBR Green PCR Master Mix (cat. no. 1725270; Bio-Rad, Hercules, CA, USA) was used, including 1.5 μl of cDNA and 0.2 μM of specific primer pairs (VEGF: forward 5′-atcttcaagccatcctgtgtgc-3′, reverse 5′-caaggcccacagggattttc-3′; PlGF: forward 5′-gttcagcccatcctgtgtct-3′, reverse 5′-ttaggagctgcatggtgaca-3′; GAPDH: forward 5′-tgtgtccgtcgtggatctga-3′, reverse 5′-cctgcttcaccaccttcttga-3′). Two-step PCR program was run: initial denaturation at 95 °C for 10 min; 40 cycles of 95 °C for 15 sec and 55 °C for 45 sec. The relative expression levels of VEGF and PlGF targeted genes were calculated using the 2^-ΔΔCq^ method and normalized to the house-keeping gene GAPDH. The fold change over the controls was presented in this study.

### Knockdown assay

To delete the expressions of VEGF and PlGF at mRNA levels, siRNA-VEGFA (sc-29520; Santa Cruz, Dallas, TX, USA) and siRNA-PlGF (sc-44027; Santa Cruz) were introduced to hRECs at the final concentration of 5, 10 or 20 nM with RNAiMAX (cat. no. 13778-075; Invitrogen; Thermo Fisher Scientific, Inc.). Non-targeted siRNA (sc-37007, Santa Cruz) was used as the controls. Cells were then collected after 48 hours. Knockdown efficiency of the siRNAs was evaluated in hRECs under normoglycemia condition **(**Supplemental Fig. 2).

### Western blot assay

As described previously^21^, western blot assay was performed. Briefly, cells were lysed in RIPA buffer (20 mM Tris-HCl pH 7.5, 150 mM NaCl, 1 mM EDTA, 1 m M EGTA, 1% NP-40, 1% sodium deoxycholate, 2.5 mM sodium pyrophosphate, 1 mM β-glycerophosphate, 1 mM Na_3_VO_4_, and 1 μg/ml leupeptin, 1 mM PMSF), and total cellular protein was extracted and quantitated. Equal amount of protein (75 μg) was separated using 7.5 or 12.5% sodium dodecyl sulfate polyacrylamide gel electrophoresis, and transferred to nitrocellulose membrane (Abcam). The membrane was blocked for 1 h in 5% low-fat milk or BSA prepared in Tris-buffered saline containing 0.1% Tween-20 (TBST), and then incubated for over-night with the indicated primary antibodies followed by incubation with HRP-conjugated goat anti-rabbit or goat anti-mouse IgG (1: 10,000; cat. no. G-21234, G-21040; Invitrogen; Thermo Fisher Scientific, Inc.) for 1 h at room temperature. After 5 washes with TBST, the blots were developed using ECL Western Blotting Substrate (cat. no. 32109; Pierce; Thermo Fisher Scientific, Inc.). To blot another Ab in the same membrane, the membrane was stripped by incubating the membrane in the stripping buffer (cat. no. 46430, Thermo Fisher Scientific, Inc.) for 15 min at room temperature; then washed three times with TBST, blocked for 1 h in 5% BSA, and another Ab was then applied for overnight at 4 °C. The specific band was detected and the intensities were quantitated by using Image J (Version 1.51 s; National Institute of Health, Rockville, MD, USA).

### PKC activity assay

PKC activity was evaluated using PKC Kinase Activity Assay Kit according to the manufacturer’s instructions (ab139437, Abcam). Briefly, 50 μg total proteins in 30 μl were added to PKC Substrate Microtiter Plate. Dilution buffer was used as Blank control. 10 μl of ATP (1 mg/ml) was added to each well, and incubated at 30 °C for 2 h. 40 μl of the Phospho-specific Substrate Antibody was added and incubated at room temperature for 1 h. After four washes, 40 μl of HRP-conjugate anti-rabbit IgG (1: 1,000) was added to each well, and incubated at room temperature for 30 min. After 4 washes, 60 μl of the TMB Substrate was added, and incubated for 45 min. Finally, 20 μl of the Stop Solution was added. The absorbance was measured at 450 nm with Microplate reader (Bio-Tec Instruments). The PKC kinase activity was expressed by the value of OD450.

### Statistical analysis

Data are shown as mean ± S.D. One-way ANOVA and Two-Way ANOVA were used to perform statistical analysis (GraphPad Prism 6.0; La Jolla, CA, USA). The value of *p* less than and/or equal to 0.05 was considered to have significant differences.

## Supplementary information

Supplementary figures
