# Supplementary material for: Distinct downstream signaling and the roles of VEGF and PlGF in high glucose-mediated injuries of human retinal endothelial cells in culture
Source: Sci Rep. 2019 Oct 25;9:15339. doi: 10.1038/s41598-019-51603-0 (PMC6814860; doi:10.1038/s41598-019-51603-0)

Distinct downstream signaling and the roles of VEGF and PlGF in high glucose-mediated injuries of human retinal endothelial cells in culture

Wanzhen Jiao[1], Jia-Fu Ji[2], Wenwen Xu[1], Wenjuan Bu[3], Yuanjie Zheng[4a,b,c,d], Aihua Ma[5*], Bojun Zhao[1*] & Qingfeng Fan[6]

Supplemental Figure 1. The identification of human retinal ndothelial cells (hRECs). Cultured hRECs were fixed with 4% PFA, and indirect immunofluorescence staining was performed for CD31 **(a)** and Von Willebrand Factor (vWF; **b)**, respectively. The absence of primary antibody was used for negative staining control **(c)**. The hRECs were identified by positive stains of CD31 and vWF under immunofluorescence microscope (100x).

Supplemental Figure 2. The evaluation of knockdown efficiency of the siRNAs in hRECs under normoglycemia condition. In normal cultured hRECs, the siRNAs were introduced at 3 different concentrations (5 nM, 10 nM, and 20 nM), respectively. Non-treated cells (CTL) and the control siRNA (siCTL, 20 nM) that does not target any human gene was used as the transfection control. 48 h after transfection, cells were lysed and total cellular protein was extracted for immunoblotting with anti-VEGF and anti-PlGF antibodies. *Data are presented mean ± SD. n = 3. *: $p < 0.05$ vs CTL.*

Supplemental Fig 3. The expression levels of Bcl-2 and the active caspase 3 were assessed in mannitol treated hRECs. hRECs were treated with mannitol (MN) for the indicated time periods. Total cellular protein was extracted, and immunoblotting assay was performed for analysis of activated caspase 3 and Bcl-2. The results show that MN treatment did not affect the levels of Bcl-2 and activated caspase 3. *3 independent experiments were performed and representative blots were provided.*

Supplemental Fig 4. Full length blots for Fig 1

Supplemental Fig 5. The effects of mannitol and Go 6976 on the Erk activation level were assessed in cultured hRECs. (a) hRECs were treated with mannitol (MN) for the indicated time periods. Cells were lysed and total protein was extracted for immunoblotting with anti-PKC, anti-phospho-Erk1/2$^{Tyr202/Tyr185}$, and anti-NOS1 antibodies. The results show that MN treatment did not

affect the levels of PKC, NOS-1 and activated Erk1/2. **(b)** hRECs were cultured for 24 h under normoglycemia condition with or without the PKC inhibitor Go 6976 (500 nM). Cells were then lysed and total protein was extracted for immunoblotting with anti-phospho-Erk1/2$^{Tyr202/Tyr185}$ and anti-Erk1/2 antibodies, respectively. The results show that Go 6976 did not affect the levels of activated Erk1/2. *3 independent experiments were performed and representative blots were provided.*

Supplemental Fig 6. Full length blots for Fig 2

Supplemental Fig 7. The effects of mannitol and SB203580 on p38MAPK and STAT1 activation levels were assessed in cultured hRECs. (a) hRECs were treated with mannitol (MN) for the indicated time periods. Cells were then lysed and total protein was extracted for immunoblotting with anti-phospho-p38MAPK$^{Tyr180/Tyr182}$ and anti-phospho-STAT1^{Ser727} antibodies. The results show that MN treatment did not affect the levels of phospho-p38MAPK and phospho-STAT1. (b) hRECs were cultured for 24 h under normoglycemia condition with or without the p38MAPK inhibitor SB203580 (10 μM). Cells were then lysed and total protein was extracted for immunoblotting with anti-phospho-p38MAPK$^{Tyr180/Tyr182}$ and anti-phospho-STAT1^{Ser727} antibodies, respectively. The results show that p38MAPK activity were significantly inhibited by SB203580, whiles SB203580 did not affect STAT1 activation level. *3 independent experiments were performed and representative blots were provided.*

Supplemental Fig 8. Full length blots for Fig 3

Supplemental Fig 9. The effects of mannitol on expressions of VEGF and PlGF were assessed in cultured hRECs. hRECs were treated with mannitol for the indicated time periods. Cells were lysed and total protein was extracted for immunoblotting with anti-PlGF and anti-VEGF antibodies, respectively. The results show that MN treatment did not affect the levels of PlGF and VEGF. *3 independent experiments were performed and representative blots were provided.*

Supplemental Fig 1

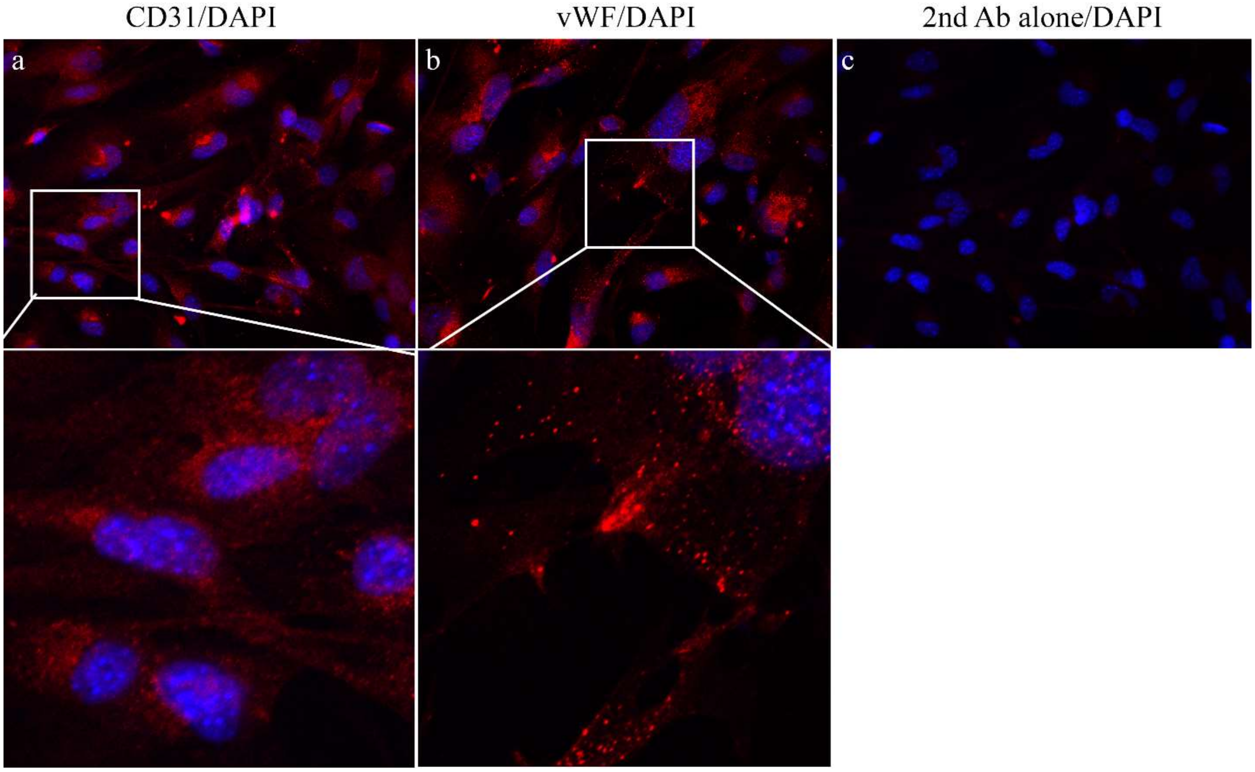

Supplemental Fig 2

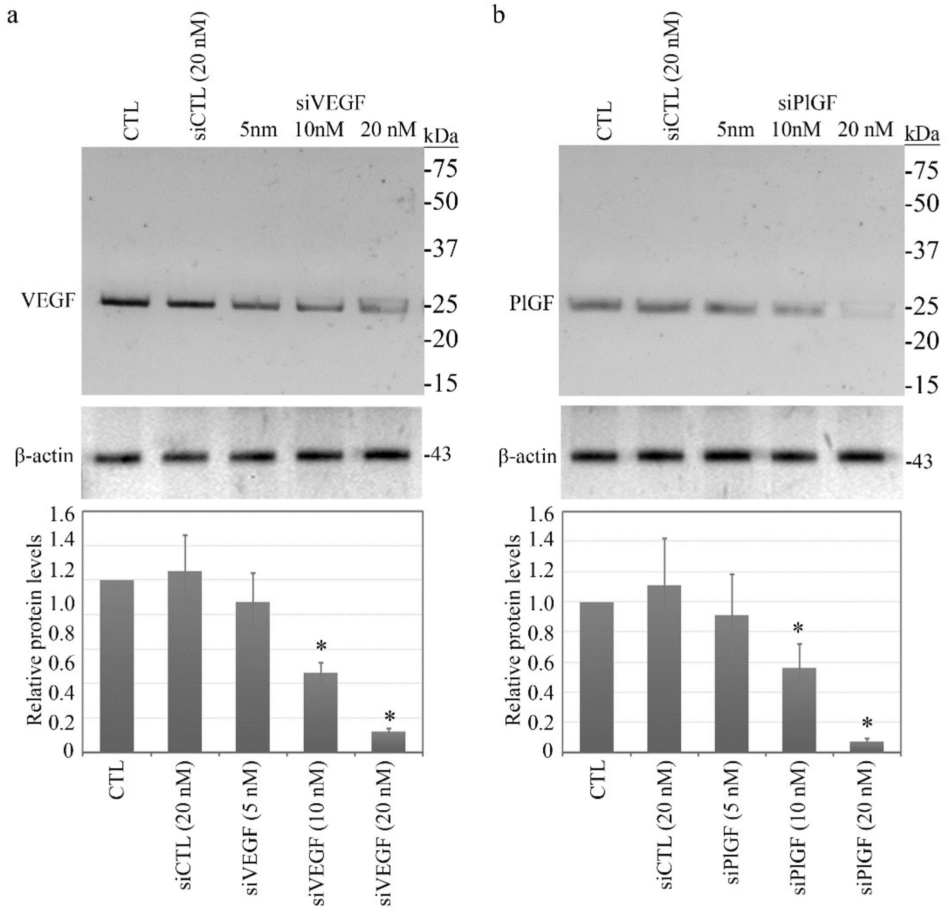

Supplemental Fig 3

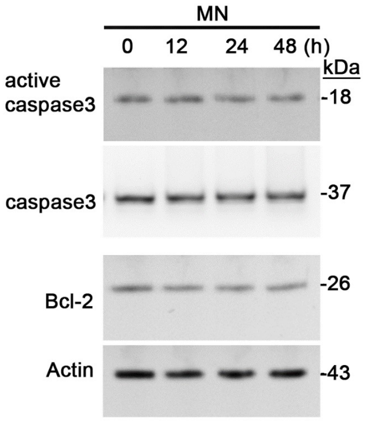

Supplemental Fig 4

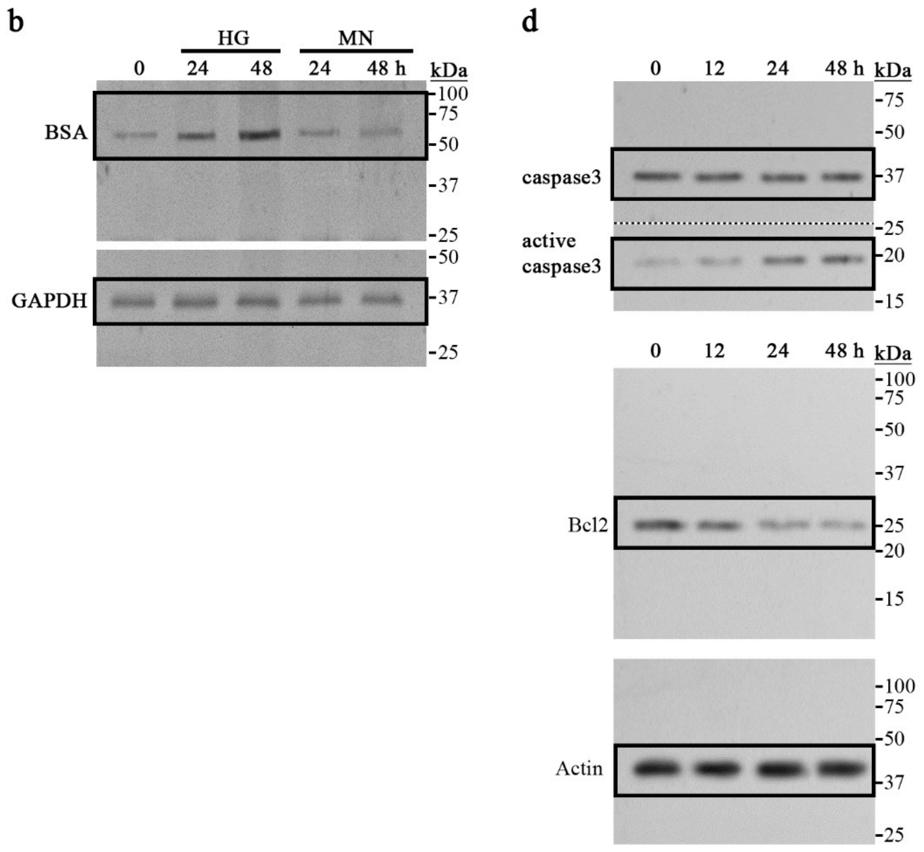

Supplemental Fig 5

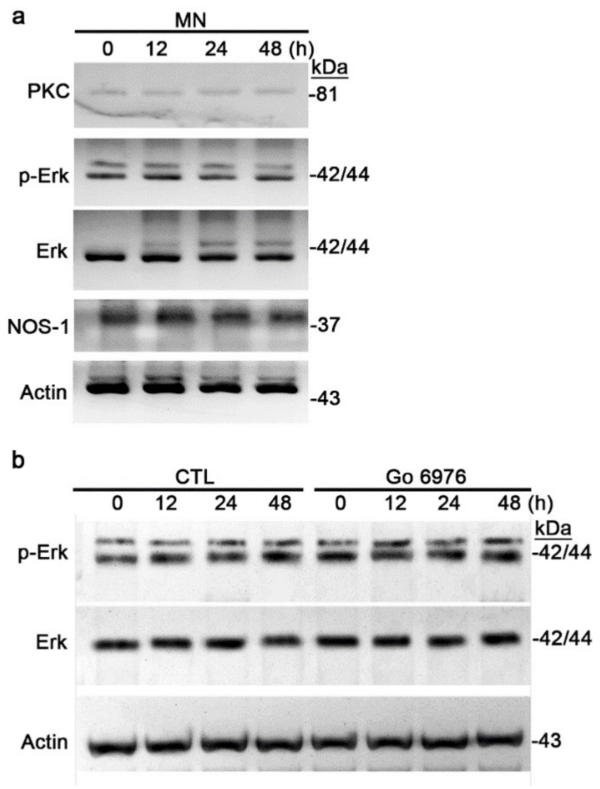

Supplemental Fig 6

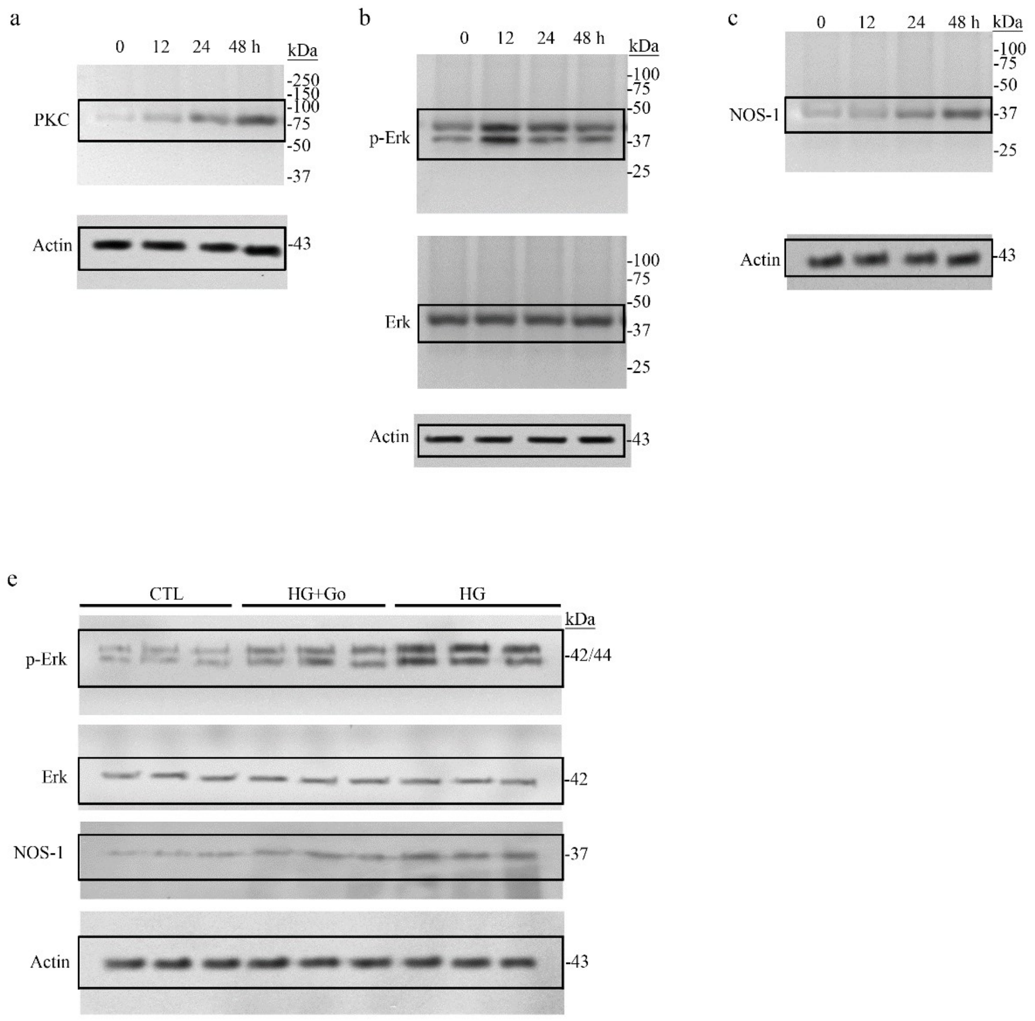

Supplemental Fig 7

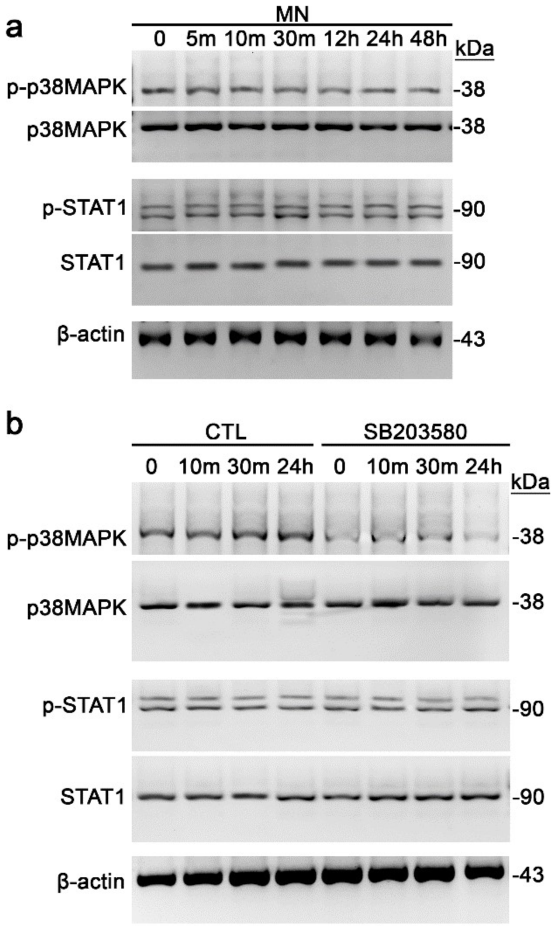

Supplemental Fig 8

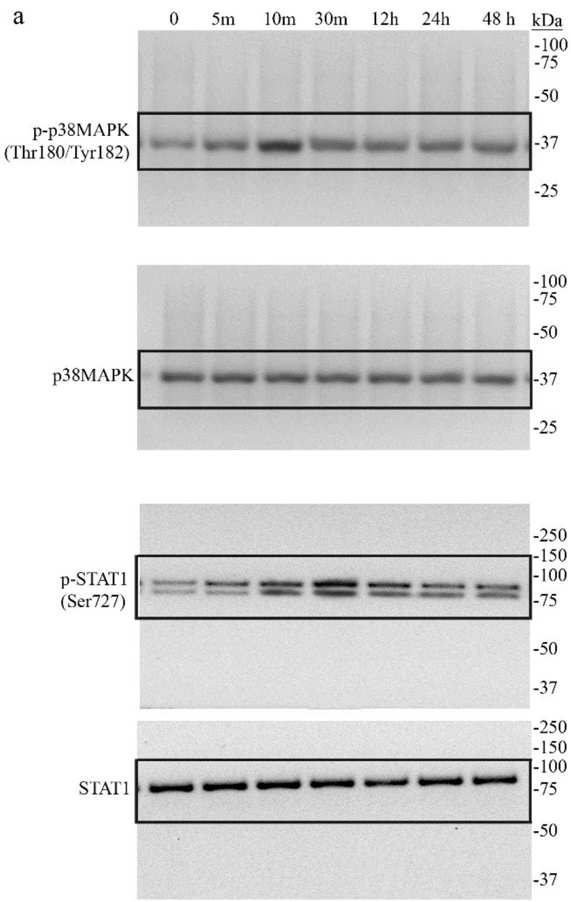

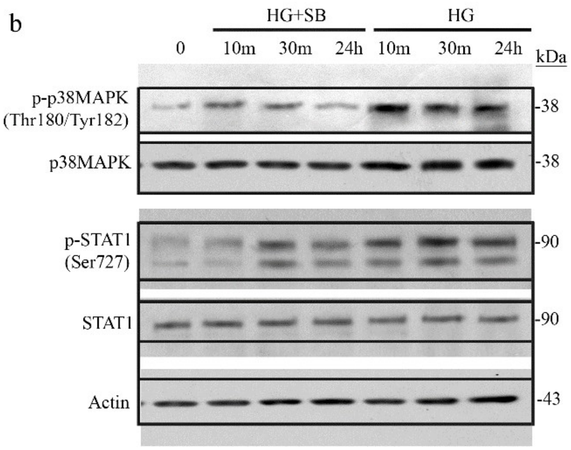

Supplemental Fig 9

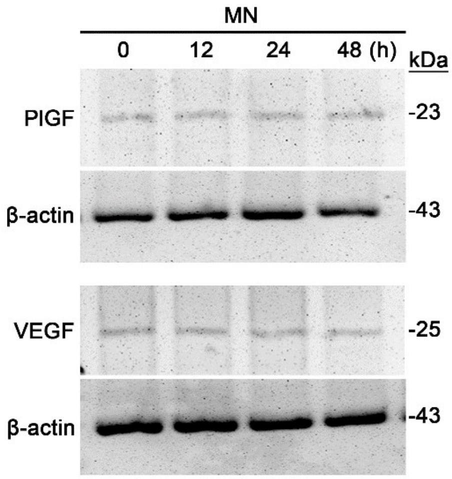

Supplement: Supplementary file 1 — Supplementary figures [file 41598_2019_51603_MOESM1_ESM.pdf]
